# Bravo® Capsule Aspiration: A Rare Case Report

**DOI:** 10.7759/cureus.1556

**Published:** 2017-08-09

**Authors:** Abdul Haseeb, Noman Lateef, Muhammad Bilal, Kumar Gaurav, Jason Prudom, Ali Musani

**Affiliations:** 1 Department of Medicine, Dow University of Health Sciences, Karachi, Pakistan; 2 Pulmonary and Critical Care Medicine, Medical College of Wisconsin; 3 Pulmonary and Critical Care Medicine, University of Colorado School of Medicine and University Hospital

**Keywords:** endoscopy, gastroesophageal reflux, ventilation

## Abstract

Bravo® capsule (BC) (Medtronic, Minneapolis, MN) endoscopy is a reliable, viable, and well-tolerated diagnostic modality for resistant gastroesophageal reflux disease (GERD). Common complications of the procedure include early dislodgment, poor transmission, and premature removal due to intractable pain, while aspiration of the capsule is exceedingly rare. This paper reports a case of BC aspiration in a 52-year-old female who presented after being ventilated when her oxygen saturation dropped. The initial chest radiograph revealed that the BC was in the right main bronchus; the site was further elaborated by flexible endoscopy and the capsule was found to be in the right lower lobe bronchus distal to the bronchus intermedius. This was followed by a rigid bronchoscopy and extraction of the capsule with rigid grasper forceps. Although this occurs rarely, immediate endotracheal intubation and ventilator support is the preferred emergency step in such cases until bronchoscopic removal can be performed.

## Introduction

The Bravo® pH Monitoring System is a catheter-free, wireless, intraesophageal pH sensing, radio-transmitting capsule. It represents the gold standard for the diagnosis of gastroesophageal reflux disease (GERD) following an unrevealing esophagogastroduodenoscopy (EGD) [1]. The capsule is adhered to esophageal mucosa transorally by endoscopy, and pH recordings are sent using radio telemetry for up to 48 hours. Previous studies have suggested that the Bravo capsule (BC) is a feasible, reliable, and well-tolerated diagnostic modality for treatment-resistant GERD [2]. The key complications of this technique include poor transmission and early dislodgment of the capsule. Removal of the capsule due to intolerable chest pain or intolerance is a relatively infrequent complication [3]. Moreover, one of the rarest, yet life-threatening, complications include aspiration of the capsule. This study reports the case of a middle-aged woman with a BC endoscopy aspiration, which was later successfully extracted. The purpose is to emphasize on the rare but potentially fatal occurrence of this condition.

## Case presentation

**Figure 1:**
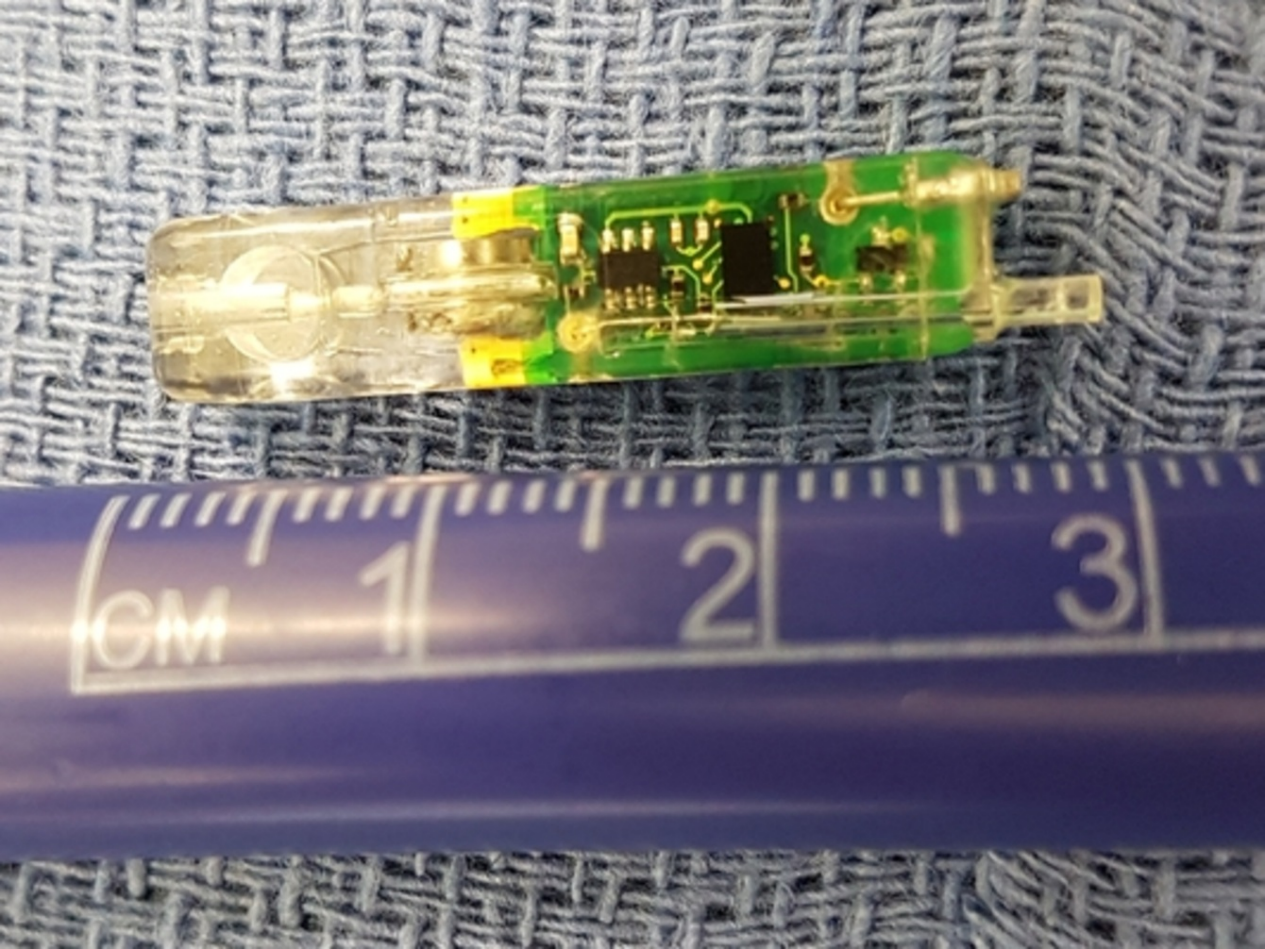
30-mm Long Bravo® Capsule

**Figure 2:**
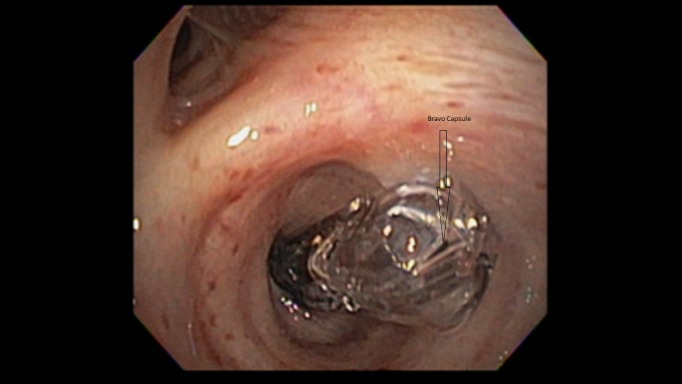
Flexible Bronchoscopy Showing Bravo® Capsule Lodged in the Right Lower Lobe Bronchus Just Distal to the Bronchus Intermedius

**Figure 3:**
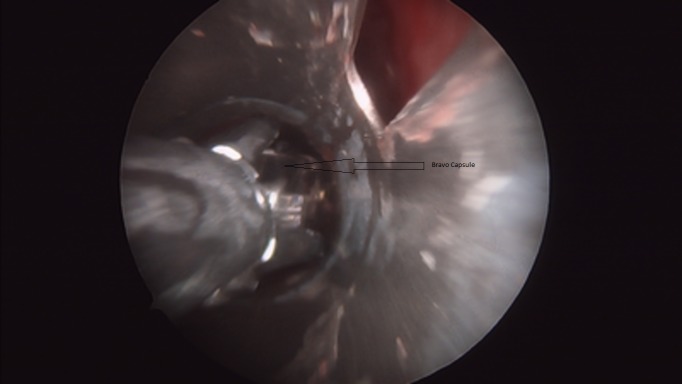
Rigid Bronchoscopy Showing Bravo® Capsule Being Extracted Using Rigid Grasping Forceps

A 52-year-old female patient with a past medical history of GERD was referred to our facility for BC extraction following an accidental aspiration during the procedure. The patient was admitted to an outside facility for a BC placement following normal EGD and gastric emptying studies. During the withdrawal of the endoscope, the capsule was dropped in the hypopharynx, which migrated further downwards after an attempt to retrieve it. The patient's oxygen saturation dropped to 74%, requiring immediate intubation. The patient remained hemodynamically stable, and she was transferred to our facility for rigid bronchoscopy and removal of the BC. The patient maintained an oxygen saturation of 95% or above on 40% FiO2 and positive end-expiratory pressure (PEEP) of 5 cm water. An initial chest radiograph revealed a 30 mm long capsule in the right main bronchus (Figure 1). The patient was immediately transferred to the operating room. Initial assessment by flexible bronchoscopy revealed the foreign body to be lodged in the right lower lobe bronchus just distal to the bronchus intermedius (Figure 2). A rigid bronchoscopy was then performed using a rigid black bronchial bronchoscope (Dumon Harrell: outer diameter, 12.5 mm) (Bryan Corp., Woburn, MA). Using rigid grasping forceps, the foreign body was secured and extracted in one piece into the rigid bronchoscope (Figure 3). Post-extraction, the foreign body was examined for any missing pieces. The airway was revisited in detail for any injuries or residual pieces of the foreign body. The patient was re-intubated with a laryngeal mask airway (LMA) 3.5 while awaiting reversal of anesthesia and muscle relaxants. The patient’s hemodynamic and respiratory status remained stable during and after the procedure. No complications were noted.

## Discussion

The use of a new wireless and catheter-free technique for pH monitoring makes the BC a widely used diagnostic modality for treatment of resistant GERD with negative EGD. It is generally considered a safe and well-tolerated procedure but with few reported technical difficulties, including non-deployment, non-attachment, misplacement, early dislodgement, and poor data transmission. However, very infrequently, more critical and potentially fatal complications can occur in fewer than 2% of the patients. These include esophageal wall trauma, excessive bleeding, and capsule aspiration [4]. The direct placement of the Bravo pH capsule under endoscopic visualization makes aspiration an even rarer event.

A comprehensive review of the literature shows only two reported cases of BC aspiration. Von Renteln, et al. presented a case of a 44-year-old patient who underwent transnasal placement of the BC, leading to aspiration into the nasopharynx [1]. The second case was reported by Kumar, et al. in which the capsule was aspirated in the pyriform fossa several hours after deployment, apparently after detaching from the esophageal mucosa [5].

Our case presents a middle-aged woman who was undergoing transoral BC placement for refractory GERD. During the deployment, the capsule remained attached to the endoscope and was inadvertently pulled up into the hypopharynx along with the endoscope. When the capsule could not be visualized in the esophagus, a thorough examination was performed and the capsule was found in the hypopharynx. The capsule then migrated into the lower airways during further attempts to remove it from the hypopharynx.

Our case is unique in its nature due to the deep aspiration of the BC into the lower lobe bronchus. Although the exact cause of deep aspiration is not known, the initial non-bronchoscopic attempt to remove it seems to be the probable cause. Immediate endotracheal intubation and ventilator support remain the mainstay of emergency treatment until bronchoscopic removal is performed. The delay in immediate intubation may increase further complications and fatality, especially in those with underlying pulmonary disease. A flexible bronchoscopy, followed by rigid bronchoscopy with rigid grasper forceps, was used to access and extract the capsule expeditiously without causing any damage to the surrounding tissues of the airways or the vocal cords.

The aspiration during capsule endoscopy, used for diagnosing occult small bowel disorders, is also rarely reported with an incidence of 0.003% [6], but it is still more common than the aspiration of a Bravo pH capsule. The main factor responsible is the different method of deployment. The capsule endoscopy uses the swallowing technique and the risk factors for capsule aspiration include old age, central nervous system depressants, poor teeth, and neurological diseases [7]. The transnasal or transoral approach using endoscopic visualization eliminates the above risk factors in Bravo pH capsule deployment.

Considering the frequent use of the Bravo pH capsule for diagnosis of GERD and the life-threatening feature of this rare complication necessitates its reporting. To avoid this rare complication and to make an early diagnosis, the following recommendations may prove beneficial [8]. They include carrying out this procedure in areas where oxygen and resuscitation facilities are available, using a real time video monitor to confirm the placement of the capsule in the esophagus, and observation of the patient in the early post-placement period.

## Conclusions

To the best of our knowledge, this is the first reported case of accidental aspiration of the BC in the lower lobe bronchus, making its removal by rigid bronchoscopy obligatory. Emergent intubation and therapeutic bronchoscopy with a rigid bronchoscope should be carried out to minimize further pulmonary complications. In the absence of a rigid bronchoscopy facility, the patient should be transferred to a nearby hospital with interventional pulmonology or thoracic surgery expertise.
